# The relationship of the psychological coping and adjustment strategies of infertile women with the success of assisted reproductive technology

**DOI:** 10.18502/ijrm.v17i2.3989

**Published:** 2019-03-20

**Authors:** Nahid Golmakani, Samira Ebrahimzadeh Zagami, Habibollah Esmaily, Atiyeh Vatanchi, Maryam Kabirian

**Affiliations:** ^1^Nursing and Midwifery Care Research Center, Mashhad University of Medical Sciences, Mashhad, Iran.; ^2^Social Determinants of Health Research Center, Mashhad University of Medical Sciences, Mashhad, Iran.; ^3^Faculty of Medicine, Mashhad University of Medical Sciences, Mashhad, Iran.; ^4^Student Research Committee,School of Nursing and Midwifery, Mashhad University of Medical Sciences, Mashhad, Iran.

**Keywords:** *Infertility*, *Psychological adjustment*, *Coping skills*, *(ART).*

## Abstract

**Background:**

The success of assisted reproductive techniques plays a very important role in the quality of life of infertile couples and decreases the negative behavior states of infertility.

**Objective:**

This study aimed at determining the relationship between psychological coping and adjustment strategies with the success of assisted reproductive technology (ART).

**Materials and Methods:**

This correlational study was conducted on 204 women visiting Milad Infertility Center in Mashhad during 2015-2016. The research instruments included Fertility Adjustment Scale and Infertility Coping Strategies Scale. The positive result of two pregnancy tests within 48 hours was considered as the success of ART.

**Results:**

The mean and standard division score of adjustment in the group achieved treatment success (34.3±8.2) exceeded the group failed (33.6±8.8), the difference was not statistically significant (p= 0.381). Also, there was no significant difference between groups in the median and interquartile range of total coping strategies 81 (13) vs. 79.5 (12.25), (p= 0.369). Based on the logistic regression model for one increased transferred embryo, the chance of getting pregnant is 1.3 times, and for each unit increase in FSH level, the chance of ART success decreases 18%.

**Conclusion:**

The results of this study showed that there is no relationship between psychological coping and adjustment strategies with ART success. However, the number of transferred fetus and tirthday FSH are introduced as factors that are related to the success of ART.

## 1. Introduction

Infertile women often consider infertility the most stressful event of their lives and describe repeated and successive treatment courses as repeated periods of crisis (1). Approximately 32% of women are at risk of developing mental health problems in the early stages of infertility treatment (2). Different infertility treatments are increasing rapidly and they are stressful for infertile women (3). The women undergoing assisted reproductive technology (ART) are often anxious and depressed due to infertility and uncertainty of treatment outcome (4). Cousineau and colleagues believed that only half of the couples seeking a solution to this problem in infertility treatment centers achieve success (5). Simbar and co-workers reported the success rate of ARTs in the infertile women visiting the selected infertility treatment centers in Tehran between 9% and 14.5% (6). The increase of unsuccessful cycles causes further physiological problems on women (7–11). Psychological problems and their associated negative behavior states, as a vicious cycle, may be a threat for the treatment consequence of Intracytoplasmic Sperm Injection (ICSI) and Intra Vitro Fertilization (IVF) (12, 13).

Different mechanisms have been proposed for the impact of negative behavior states on infertility including impairing the secretion of gonadotropins, local effect of catecholamine on uterus and function of fallopian tubes, and impairing the immunological trends participating in the preservation of fertility and implantation (14). The psychological aspects of adjustment to and coping with infertility were introduced as the major factors effective in the psychological pressures caused by infertility. Adjustment to infertility is a mental processing method by which an individual processes the information related to the problems caused by infertility, therapeutic interventions, and their outcomes (15–18). Coping strategies encompass a group of behaviors exhibited by an individual while encountering the problems caused by infertility (19). Studies show that infertile couples apply a wide range of methods of coping with infertility (20). Different ways of coping with infertility include avoidance of infertility experience reminders, hide or discharge of inner feelings, self-blame, denial, and appeal to religious beliefs (21).

Different studies have proved the relationship between physical–mental problems and infertility (22, 23); however, various aspects of infertility are still unknown (24). With respect to the role of mental pressures on the outcome of ART, it is hoped that the stresses caused by long-term and time-consuming treatments and extremely high treatment costs, especially when they hinder treatment, are avoided through identification and intervention of the factors related to ART success.

Therefore, a study was conducted aiming at determining the relationship between different strategies of psychological coping and adjustment of infertile women and ART.

## 2. Materials and Methods

This correlational study was conducted on 204 infertile women visiting the Milad Infertility Center in Mashhad during 2015-2016. The sample size was estimated as 200 individuals using the result of a pilot test based on the correlation formula and with respect to the confidence coefficient of 95% and the power of test of 80%. Two hundred and twenty individuals were included by assuming the probability of 10% exclusion. Sampling was performed using non-probabilistic accessible method. After the initial evaluations of the inclusion criteria for the participants who were candidates for ART, they were helped to fill out the profile form provided by the research unit. Inclusion criteria included having the ability to read and write, consuming no drugs affecting psyche, having no record of certain mental disorders and/or hospitalizing in mental hospitals, experiencing no adverse or stressful record such as the death of first-degree relatives, family severe disorders, financial problems, major change in living conditions in the past six months. In the next step, the women were requested to complete Fertility Adjustment Scale (FAS) and Infertility Coping Strategies. The scale of infertility coping strategies includes a modified form and a combination of two instruments for coping strategies in the study of Pottinger and co-workers and Donkor and Sandall (19, 20), which includes five domains of denial, talking to others, taking control, passing as normal, attributing a problem to fate, and 30 phrases with a 4-point Likert scales (never= 1, sometimes= 2, usually= 3, always= 4) with the range of 30–120. Validity was determined using content validity and reliability was determined (a= 0.75) using internal consistency by calculating Cronbach's alpha. FAS was designed by Glover and co-workers (15) in the Department of Mental Health in London in 1998 and it has been used since then as a valid and reliable tool. It includes 12 phrases with a 5-point Likert scale (1= strongly disagree, 2= somewhat disagree, 3= neutral, 4= somewhat agree, 5= strongly agree) within the range of 12–60. Validity was determined using content validity and reliability was determined (r= 0.70) using the test–retest method.

The form of medical records of the participants was filled out by the researcher using the checklist of patient medical records to study the ART success. This way, the positive result of *Beta*-Human Chorionic Gonadotropins test and its repetition within 48 hr with an elevated titer compared to the first test was recorded in the checklist as the treatment success, and the negative *Beta*-Human Chorionic Gonadotropins test was recorded as the treatment failure.

### Ethical consideration

Samples were taken after obtaining the confirmation of the committee of ethics of the university, obtaining written consent from participants, and taking the code of ethics (Code: 6096094).

### Statistical analysis 

The statistical analysis was performed in SPSS software (Statistical Package for the Social Sciences, version 16.0, SPSS Inc, Chicago, Illinois, USA). Descriptive statistics including mean, standard deviation, and frequency distribution were used for data description. The Chi-square test or the Fischer's exact test, independent *t*-test and Mann–Whitney test were used to analyze the data for comparing the two groups. The relationship between the coping and adjustment strategies of the infertile women and ART success was examined using logistic regression model. The mean difference is significant at the 0.05 level (p< 0.05).

## 3. Results

The mean age of infertile women was 29.9±5.5. The mean duration of infertility was 6.6±4.5, and they have been undergoing infertility treatment for 2.8±3.0 yr; 21% women were employed. Among them, 64.7% were teachers or had a cultural occupation. High school diploma was the highest percentage of level of education in the participants (40.2%) and their spouses (28.4%). The mean age of spouses was 33.7±6 yr within the range of 20–62 yr and 54.4% were self-employed. In response to the question on the economic problems caused by the treatment costs for infertility, more than 50% of women (53.9%) chose `high' or `very high' options. Table I shows the infertility data. In this study, 22.5% of participants achieved ART success. Chi-square test results showed that in the two groups (success vs. failure of ART), there was no statistically significant difference in the level of education and occupation, the level of education and work of the spouse, the history of previous pregnancy, previous history of ART, and the type of treatment process (p= 0.126). But economic problems were significantly higher in unsuccessful than successful group (p= 0.048) and the two groups had a significant difference in the infertility causes (p= 0.069) so that the male infertility was more in the successful group. Also, based on Fisher's exact test results, the proportion of women who had a history of ART failure was significantly higher in the unsuccessful group (p= 0.032). The variables such as age, spouse's age, duration of life together, and duration of infertility treatment did not show a significant difference between the two groups. Only the group that achieved treatment success had a shorter duration of infertility (p= 0.029) and more average rating of the number of transferred embryos (p= 0.048). In order to compare the mean FSH level of the third day of the cycle, the results of independent *t*-test showed a significant decrease in the successful group (p= 0.013). Analysis of coping strategies scale shows that the overall mean score of coping in infertile women is 81.2±10.8 in the range of 50–108. The most coping strategies of the infertile women for coping with their infertility problem include belief in miracles (76%), belief in God's will (72.5%), hope (71.1%), and attempt to achieve the recommendations of their physician (71.1%). The lowest coping strategies never selected by the infertile women included obtaining information from the Internet (52%) and receiving advice from others (50.5%). To compare the two groups in terms of different coping domains, the results of the Mann–Whitney test are shown in Table II. In this research, the mean score of FAS is 34.1±8.3 within the range of 15–55. Among the questionnaire items, the choice `strongly disagree' gained the highest percentage (41.2%) for `I feel that I only breathe and apparently I am alive'. The choice `strongly agree' gained the highest percentage (68.6%) for `more than anything else in the world, I would like to have my own child'. The independent *t*-test shows that although the mean score of adjustment in the group achieved treatment success (34.3±8.2) exceeded, the group failed to achieve fertility (33.6±8.8), the difference was not statistically significant (p= 0.381). Finally, based on the logistic regression model for one increased transferred embryo, the chance of getting pregnant is 1.3 times and for each unit increase in FSH level, the chance of ART success decreases 18% (Table III).

**Table 1:** Infertility characteristics of the infertile women.


**Characteristics of Infertility**	**Value**
Common lifetime (yr)${}^{*}$	7.9 ± 4.8
Duration of infertility (yr)*	6.6 ± 4.5
Duration of infertility treatment (yr)*	2.8 ± 3.0
Factors of infertility**	
Male infertility	65 (31.9)
Female infertility	62 (30.4)
Both of them	(17.2) 35
Unknown	42 (20.5)
Previous kinds of ART**	
IUI	(61.2) 125
IVF	(24.3) 50
IUI & IVF	(6.8) 14
Previous experience of ART	(50) 102
Previous failure of ART	(91.8) 187
Note: *Data were described by Mean ± SD; **Data were described by *n* (%); ART: Assisted Reproductive Technology; IUI: Intra Uterine Insemination; IVF: Intra Vitro Fertilization.	

**Table 2:** Comparison of the coping strategies in success vs. failure after ART treatment in the infertile women.


	**Success Group (n= 46)**	**Failure Group (n= 158)**	**p-value**
Denial	16 (6)	15 (4.25)	0.162
Talking	11 (5)	11 (6)	0.331
Control	27 (6)	25 (7)	0.154
Passing	7 (3)	7 (2.25)	0.09
Fate	18 (4)	17 (5)	0.037
total	81 (13)	79.5 (12.25)	0.369
Note: *Data were described by Median (Interquartile Range) and analyzed by Mann–Whitney test.			

**Table 3:** The association of infertility related factors with successful ART based on logistic regression model.


	**df**	**OR**	**CI 95%**	**p-value**
Infertility adjustment	1	1.36	0.721-1.58	0.563
Coping with Infertility	1	1.04	0.623-1.20	0.283
Duration of infertility	1	0.35	0.119-1.95	0.438
Number of transferred Fetus	1	1.32	1.18-2.10	0.005
Previous Failure of ART	1	0.712	0.501-2.41	0.54
Tirthday FSH	1	0.821	0.671-0.930	0.032
Note: *Data were analyzed by logistic regression model; ART: Assisted Reproductive Technology; FSH: Follicle Stimulating Hormone; OR: Odd Ratio; CI: Confidence Interval.				

## 4. Discussion

The results of this study showed that there is no relationship between coping skills and adjustment strategies with ART success. In this study, 22.5% of the participants achieved a positive result from a pregnancy test. The rate is 17–39% in Milad Research Center in Mashhad. A success rate of 35% was reported for ART in Iran (the head of the Iranian Society of Fertility and Infertility) (25). This study showed no statistically significant relationship between the coping score and age, married life duration, infertility treatment duration, and the type of treatment method (p> 0.05), which is consistent with the study of Glover and colleagues. The study of Glover and co-workers in London aimed at measuring FAS. The mean scores of the psychological adjustment of infertile women in two studies were close to each other (15). In this study, the maximum coping strategies of the infertile women included `relying on religious beliefs' and `attempt to achieve the recommendations of physician', and the minimum coping strategies were `receiving advice from others' and `obtaining information from the Internet'; these findings are consistent with those of Donkor and Sandall, Pottinger and co-workers (19, 20). In the study of Donkor and Sandall, `Fatalism' and `Reliance on religious beliefs' were used most in the infertility coping strategies. This way, the choice of the majority of women was very much in response to `I believe that it is the act of God and if He deemed appropriate, I would be pregnant' and the choice of 95% of the women in response to `I pray' and `I hope for a miracle to happen' was `very much'. The minimum coping strategy was the expression of the inner feelings of the infertile individuals to others, as 66% of them would never express their inner feelings to others (19). Pottinger and co-workers showed that the highest and the lowest frequent coping strategies among infertile couples were `attempt to achieve the recommendations of physician' and `avoid meeting pregnant women or family members with small children' (20). Respectively, this study showed no statistically significant relationship between different types of psychological coping and adjustment strategies and ART success, which is somehow consistent with the study of Simbar and colleagues. In the study of Simbar, researchers concluded that although consultation and the reduction of the anxiety level of infertile women was necessary for improving their quality of life, there was no relationship between the anxiety level and ART result (6). On the other hand, Kraaij and co-workers showed that Cognitive coping skills seemed to have a stronger influence on the effect than the behavioral coping skills. Also, adjusting the goal to have children seemed to be a helpful way to cope. These findings suggested that intervention programs should pay attention to both cognitive coping skills and goal adjustment (26). Despite cultural differences among countries, the results of different studies indicate a relative coordination between the mean score of coping with infertility and various infertility adjustment strategies, which may express the existence of common feeling among infertile women. Study limitations included the selection of participants from a training infertility center. The coping score in the group that achieved success was higher; therefore, further studies with greater sample size are recommended.

In the end, with regards to the multifactorial nature of the success of ART and the impossibility of controlling all interventional variables in this field, it seems that carrying out correlational studies with more sample size or conducting interventional studies can be used to examine the impact of various coping and adjustment strategies on the success of infertility treatment methods will be more helpful.

## 5. Conclusion

The results of this study showed that there is no relationship between psychological coping and adjustment strategies with ART success. However, the number of transferred fetus and tirthday FSH are introduced as factors that are related to success of ART.

##  Conflicts of Interest

The authors declare that there is no conflict of interest to declare in this study.
